# Understanding clinician barriers to providing equitable healthcare for ethnic minority populations

**DOI:** 10.1093/eurpub/ckac131.522

**Published:** 2022-10-25

**Authors:** M Patel, R Newell, S George, H Clark

**Affiliations:** Newham University Hospital, Barts Health NHS Trust, London, UK

## Abstract

**Background:**

It has long been recognised that ethnic minority groups have worse health outcomes in the UK. Social determinants of health (SDH) contribute significantly to these inequalities. However, inequalities persist, even after controlling for these determinants. As well as being less likely to engage with healthcare services, ethnic minority groups are more likely to report poor experiences. The majority of healthcare in the UK is delivered through patient: clinician interactions (PCI), therefore a good working relationship with patients is paramount. Recent focus has been placed on ensuring “cultural competence”. Whilst this is important, we suggest also examining the culture within healthcare itself. Healthcare professionals are not immune to bias, preconceptions and the stresses of work and this must be taken into account. A seminal piece of work on this is the “culture of medicine” framework proposed by Boutin-Foster et al, which examines the impact of these factors.

**Aims and Methodology:**

This paper aims to examine barriers within PCI that impact healthcare for ethnic minorities. A formal literature review was conducted and 131 relevant studies were identified. Grounded theory was used for analysis and data was categorised into themes with Boutin-Foster’s framework used as a structure.

**Results:**

The review found that the concept of implicit bias was paramount in PCI. Three major barriers resulting from this bias are suggested: its impact on clinical decision making, the impact on clinician-patient communication and finally the resultant lack of trust in clinicians and poor perceived quality of care by ethnic minority groups.

**Conclusions:**

Clinician implicit bias is a major barrier to equitable healthcare for ethnic minority populations. A solution we propose is to acknowledge our own preconceptions. Awareness of our own culture, preconceptions and the pressures around us will allow us to find solutions to these barriers, including further research and education.

**Key messages:**

• Clinician implicit bias within the “Culture of Medicine” is a barrier to equitable healthcare for ethnic minority populations.

• Awareness of our own culture and preconceptions is paramount to addressing these barriers.

